# Knot formation and spread along the shoot stem in 13 olive cultivars inoculated with an indigenous pathobiome of 7 species of *Pseudomonas* including *Pseudomonas savastanoi*

**DOI:** 10.1371/journal.pone.0289875

**Published:** 2023-08-11

**Authors:** Matteo Zucchini, Antonietta Maoloni, Enrico Maria Lodolini, Ilario Ferrocino, Lucia Aquilanti, Davide Neri

**Affiliations:** 1 Dipartimento di Scienze Agrarie, Alimentari e Ambientali, Università Politecnica delle Marche, Ancona, Italy; 2 Council for Agricultural Research and Economics, Research Centre for Olive, Fruit and Citrus Crops, Rome, Italy; 3 Department of Agricultural, Forest, and Food Science, University of Turin, Torino, Italy; University of Salento Department of Biological and Environmental Sciences and Technologies: Universita del Salento Dipartimento di Scienze e Tecnologie Biologiche ed Ambientali, ITALY

## Abstract

Olive knot is a widely spread disease among olive (*Olea europaea* L.) trees. *Pseudomonas savastanoi* pv. *savastanoi* is recognized as the primary causative agent of the disease however, recent evidence indicated that consortia of bacteria (pathobiome), may favor its development. Several factors are involved in the host-plant relationship and affect the intensity of the symptoms. Among these the presence of wounds, or damages to the plants’ tissues may affect the intensity and propagation of the disease. It remains unknown whether or not bacteria move from an infected wound to another not infected one via shoot tissues. The present investigation focused on the susceptibility to olive knot of several cultivars after inoculating artificial wounds with selected *Pseudomonas* species, while spreading the disease from these to wounds on the same stem, that had not been purposefully inoculated. The pathobiome for the inoculum was prepared with 7 species of *Pseudomonas* (including *Pseudomonas savastanoi* pv. *savastanoi*), isolated from knot samples collected from two different, heavily infected olive orchards. The inoculation was done after the manual execution of 10 horizontal wounds on the stem of potted plants of 13 olive cultivars grown in the greenhouse. Only the lowest 5 wounds were inoculated. The inoculated wounds showed a maximum percentage of knots after 187 days. All 13 cultivars showed knots yet, the cultivar with the most severe disease level to *Pseudomonas savastanoi* pv. *savastanoi* was ‘Rosciola colli Esini’. The metataxonomic analysis performed on the olive knots removed after 225 days confirmed the dominance of the inoculated species *Pseudomonas savastanoi* in all the assayed cultivars. The not inoculated wounds did not show the knot disease likely because the bacterium’s inability to transmigrate from the inoculated wounds to the non-inoculated ones.

## Introduction

Olive knot is a well-known pathology of olive (*Olea europaea* L.) trees and its symptoms can be easily recognized by tumors (or galls), growing on the woody tissues or, sometimes on roots, leaves and/or fruits [1–3]. Although the Gram-negative bacterium *Pseudomonas savastanoi* pv. *savastanoi* (herein referred to as Pss) has been recognized as the primary agent of olive knot disease [4], in the recent years evidence has emerged to suggest that a consortium of bacteria (pathobiome) may favor the development of the disease [5–9]. In addition, Pss can affect several other species [10–13] including oleander (*Nerium oleander* L.), pomegranate (*Punica granatum* L.) and myrtle (*Myrtus communis* L.).

Several factors are involved in the host-pathogen relationship thus, affecting the intensity of disease manifestations. The interaction among the bacteria of the pathobiome with Pss can affect the development of the knots [14], whereas the cultivar of the host species can influence the diversity of the pathobiome population [15].

Another key factor is the intensity of damages and injuries in the tissues of the host plant. Pss enters the host through wounds, stimulating the infection process through a series of reactions that lead to knots developments through an activation of hrp genes and secretion of phytohormones via the hypersensitive response reaction [16].

The wounds triggering the infection in the host’s tissues can be caused by natural and mechanical-physical events, some of which are attributable to harvest, pruning, hailstorms and, as studied by Valverde et al. [17], by late winter frost damages. Although it is not clear the importance of tolerating late frost damages compared to those caused by the bacterium, it has been demonstrated that frost events in late winter can affect the intensity and severity of the disease in different cultivars [17, 18].

Environmental conditions remain important to determine the success of an infection outbreak, or the time of expression of specific symptoms, since the penetration of Pss in the host plant tissues. In fact, the knots can appear in a period ranging from two weeks to several months after inoculation, according to temperature and humidity conditions [19]. Yet, the literature is still unclear about reporting Pss mobility through the trees’ stem.

Various researchers have analyzed the susceptibility of olive cultivars to Pss under controlled environmental conditions. For example, Varvaro and Surico [18] found different responses in the time of disease appearance during 90 days since an artificial inoculation of Pss in 6 cultivars, where ‘Nocellara del Belice’ and a ‘wild olive’ trees showed 100% of the inoculated wounds with knots as early as 8 days since inoculation. Cultivars ‘Leccino’ and ‘Frantoio’ showed knots on 85% of the inoculated wounds, whereas knots appeared only on the 18% of the inoculated wounds in trees of the ‘Coratina’ cultivar. Benjama [20] found different behaviors among different cultivars and different bacterial strains with ‘Frantoio’ being slightly more susceptible than ‘Ascolana Dura’. Penyalver et al. [1] reported that ‘Arbequina’ was a highly susceptible cultivar to two different Pss strains whereas, ‘Ascolana Tenera’ presented a variable susceptibility depending on the strain, and ‘FS-17’^®^ showed low susceptibility to both Pss strains. In a field evaluation, Salaman et al. [21] found that susceptibility to olive knot varied significantly among cultivars and environments.

The purpose of this study was to assess the olive knot formation in 13 olive cultivars (international, Italian, including autochthonous varieties from the Marche region), after controlled inoculations with an olive knot (indigenous pathobiome of 7 Pseudomonas species, including Pss), of intentionally-made wounds and the knot propagation along the stem, from inoculated to not inoculated wounds.

## Materials and methods

### Isolation of *Pseudomonas* spp.

In September 2018, knot samples were taken from different olive trees in two orchards where several local and international cultivars were grown. The first orchard is at the Polytechnic University of Marche experimental farm “Pasquale Rosati” (AN, Italy), which is located at: 43°35’21.1 "N; 13°17’22.8"E. The second orchard is located at Monte San Vito (AN, Italy), at: 43°32’29.6"N; 13°22’38.3"E. Trees in both the orchards were heavily affected by olive knot as a consequence of the late frost, which occurred in February 2018 [17]. To isolate *Pseudomonas* spp. for the preparation of an inoculum containing *Pseudomonas savastanoi*, the knot samples were randomly taken from ‘Frantoio’, ‘FS-17®’, ‘Piantone di Mogliano’, ‘Leccino’, ‘Maurino’, ‘Piantone di Falerone’, ‘Rosciola Colli Esini’, ‘Ascolana Tenera’, and ‘Carboncella’ cultivars.

The sampled olive knots were transported to the laboratory under refrigerated conditions and stored at 4°C until use. Olive knots were homogenized using a sterile mortar in a sterile 0.85% (w v^-1^) NaCl saline solution. Two different samples were made according to the orchard of origin. Ten-fold serial dilutions were prepared in sterile physiological solution (NaCl 0.85%, w v^-1^) and aliquots (100 μL) of each dilution were spread onto *Pseudomonas* agar base (VWR, International, Radnor, PA, USA), supplemented with *Pseudomonas* CFC selective supplement (VWR) and incubated at 30°C for 48 h.

Thirty well separated colonies were randomly picked from the plates and streaked to purity for three times onto the same selective growing medium. Pure cultures were stored in Luria Bertani broth (LB) (tryptone 10 g L^-1^, NaCl 10 g L^-1^, yeast extract 5 g L^-1^) added with 25% (v v^-1^) glycerol.

### Identification of the pure cultures

The 30 presumptive *Pseudomonas* isolates underwent a preliminary Gram staining followed by DNA extraction using the method described by Hynes et al. [22] with slight modifications as reported by Osimani et al. [23]. DNA quantity and purity of the extracts were assessed spectrophotometrically, as previously described [23]. Molecular identification of the isolates was accomplished by sequencing the 16S rRNA gene with the primers 27f (5’-GAG AGT TTG ATC CTG GCT CAG-3’) and 1495r (5’-CTA CGG CTA CCT TGT TAC GA-3’) [24]. In detail, 3 μl (corresponding to ~ 100 ng of bacterial DNA) of each extract was amplified in a reaction volume of 50 μL, containing 1 U of Taq polymerase (SibEnzyme Ltd, Novosibirsk, Russia), 1 × reaction buffer (60 mM Tris-HCl, 1.5 mM MgCl2, 25 mM KCl, 10 mM 2-mercaptoethanol, 0.1% Triton X-100), 0.2 mM of dNTPs, and 0.2 μM of each primer. Polymerase Chain Reaction (PCR) was performed in a thermal cycler (My Cycler, Bio-Rad Laboratories, Hercules, USA), using the following cycling program: initial denaturation at 95°C for 5 min, followed by 35 cycles of denaturation at 94°C for 1 min, annealing at 55°C for 1 min and extension at 72°C for 2 min, and final extension at 72°C for 15 min. The PCR products were sent to Genewiz Europe (Leipzig, Germany) for sequencing. The isolates were then identified by aligning the obtained sequences to the sequences deposited at the GenBank database (http://www.ncbi.nlm.nih.gov/) using the BLAST algorithm [25]. The sequences of the 30 newly identified isolates are available at the GenBank DNA database of NCBI (accession numbers OQ990727-OQ990756) as detailed in Table 3.

### Preparation of the inoculum

With reference to recent research works [8, 9, 14], which showed how the pathobiome interaction in the olive knot contributes to form bigger knots, a consortium of 7 different *Pseudomonas* species was formulated to inoculate the wounds (Table 1).

**Table 1:** *Pseudomonas* isolates selected for the formulation of the inoculum.

Field of isolation	Isolate cod.	Species
Experimental farm “Pasquale Rosati”	AGU2	*Pseudomonas coleopterorum*
	AGU9	*Pseudomonas caspiana*
	AGU10	*Pseudomonas caspiana*
	AGU15	*Pseudomonas graminis*
	AGU18	*Pseudomonas lutea*
Orchard located at Monte San Vito	SAN1	*Pseudomonas savastanoi*
	SAN13	*Pseudomonas harudinis*
	SAN16	*Pseudomonas bohemica*

More in detail, 8 isolates (one for each identified species and one more for the most represented species *Pseudomonas caspiana*) were selected and used, in equivalent proportions, for the preparation of the inoculum. Each isolate was sub-cultured twice, with an initial 3% (v v-1) inoculation in LB broth incubated at 30°C for 48 h. At the end of the incubation period, the bacterial load of each microbial suspension was determined spectrophotometrically, with readings at 600 nm (OD600 = 0.9 = 1 * 10^9^ CFU mL^-1^) [2]. Then, aliquots containing 8 Log CFU mL^-1^ were collected from each microbial suspension, mixed, and centrifuged at 4.000 rpm for 10 min; the obtained cell pellets were re-suspended in sterile physiological solution to reach a final load for each isolate, of 8 Log CFU mL^-1^.

### Experimental design and plant material

Before starting some plantlets were removed because they were not conforming to the standard, hence the experiment was represented by a complete randomized design formed by different numbers of trees basing on the cultivar.

Self-rooted, two-year-old plotted olive plants were used for the cultivars ‘Rosciola Colli Esini’, ‘Ascolana Tenera’, ‘Ascolana Dura’, ‘Carboncella’, ‘Piantone di Mogliano (A)’ and ‘Maurino (A)’. The average of the Stem Cross Sectional Area (TCSA) of these olives was 46.0±12.9 mm^2^ on first internode.

Self-rooted, one-year-old potted olive plants were used for the cultivars ‘Frantoio’, ‘FS-17®’, ‘Piantone di Mogliano (B)’, ‘Leccino’, ‘Leccio del Corno’, ‘Pendolino’, ‘Maurino (B)’, ‘Arbequina’ and ‘Piantone di Falerone’. The average of the TCSA of these olives was 20.4±9.6 mm^2^ on first internode.

‘Maurino A’ and ‘Piantone di Mogliano A’ differ from ‘Maurino B’ and ‘Piantone di Mogliano B’ not only for the age of the plants, but also for the nursery provenience.

The experimental layout was a complete randomized design formed by: 10 plants of ‘Ascolana Tenera’, ‘Leccino’, ‘Leccio del Corno’, ‘Pendolino’ and ‘Piantone di Mogliano B’ with 6 inoculated plants and 4 control (not inoculated plants); and 15 plants of ‘Arbequina’, ‘Ascolana Dura’, ‘Carboncella’, ‘Frantoio’, ‘Fs-17®’, ‘Maurino A’, ‘Maurino B’, ‘Piantone di Falerone’, ‘Piantone di Mogliano A’, ‘Rosciola colli Esini’ with 10 inoculated plants and 5 control (not inoculated) plants.

**Fig 1:**
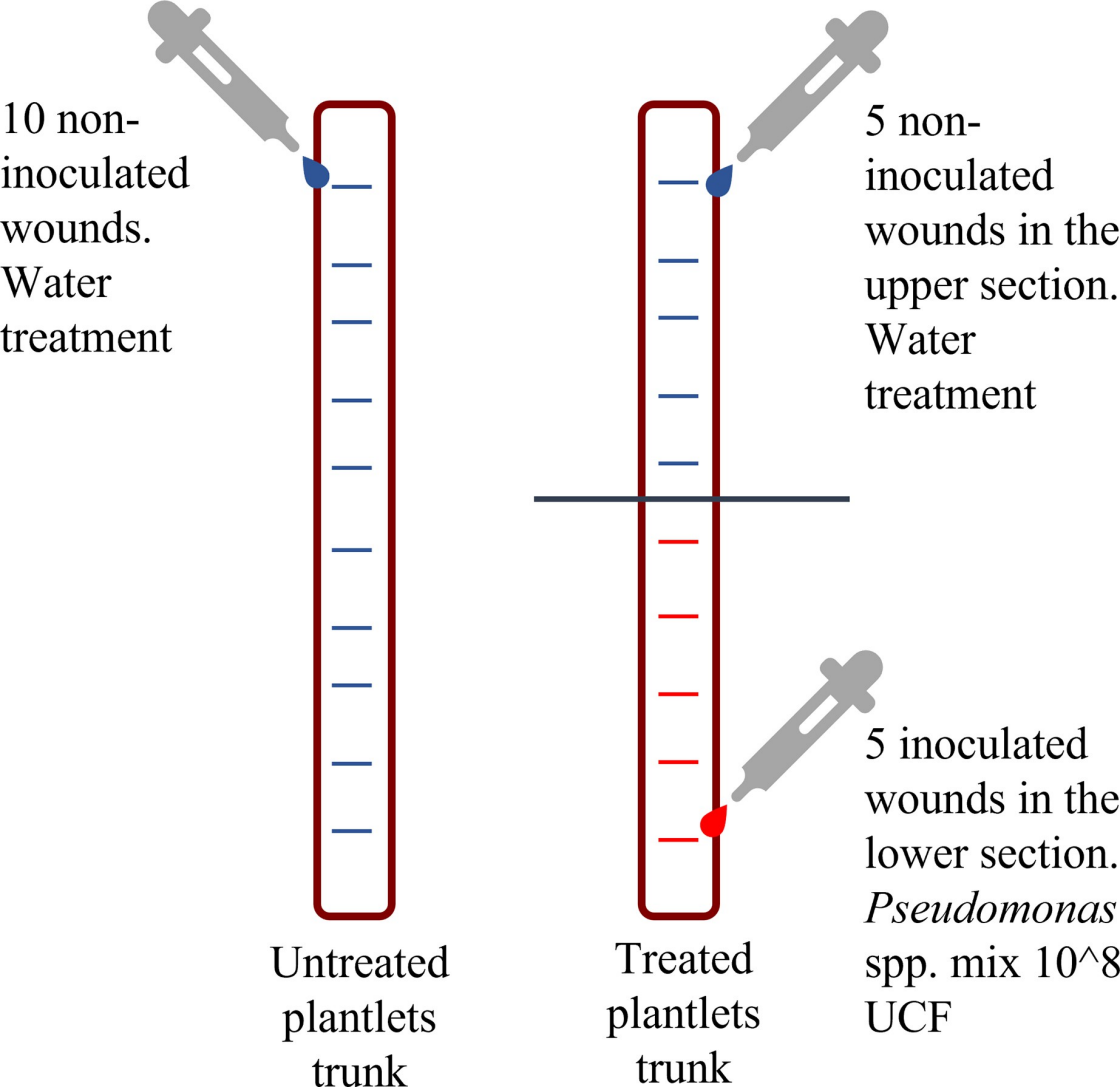
Design of experiment on plants.

In all plants, the wounds consisted in 10 horizontal 5 mm long cuts (about one per internode), that were manually executed on the bark of the stem using a sterile surgical scalpel. On the inoculated plants the first 5 wounds made in the lower section of the stem were inoculated with aliquots (10 μL) of the *Pseudomonas* spp. suspension (~8 * 10^8^ CFU mL^-1^), prepared with the isolates listed in Table 1. The 5 wounds in the upper section of the stem of the inoculated trees were treated with distilled water as it was done in all the 10 wounds made on control plants (Fig 1).

The experimental trial was executed in a heated greenhouse (fixed minimum 18°C) and began on the 3^rd^ of March 2019.

### Observations and measurements

**Fig 2:**
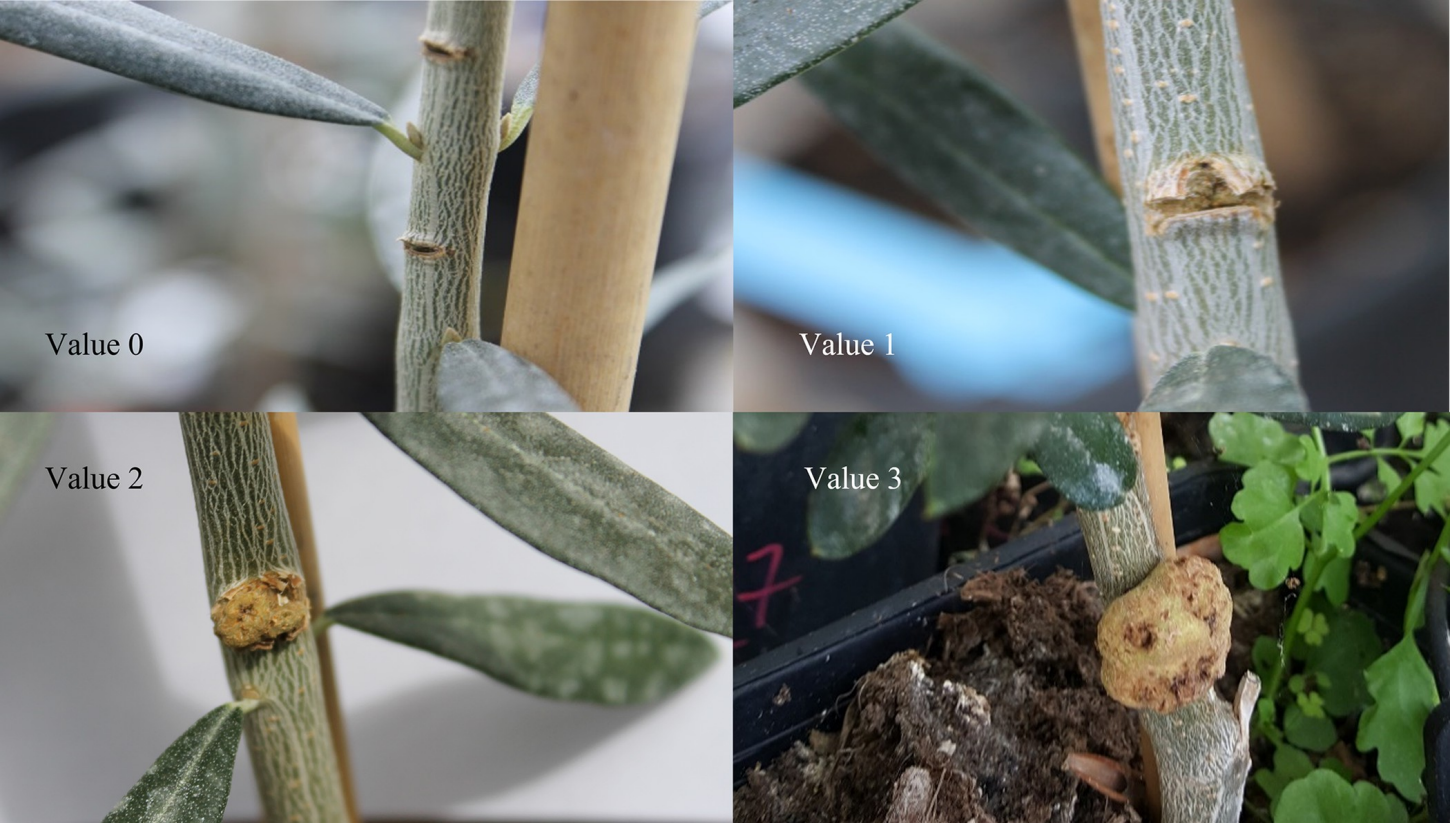
Visual index for the formed knot attributed to each wound: Value 0 = absence of knot, value 1 = the presence of knot was doubtful, value 2 = visible knot, value 3 = big and well-defined knot.

From DAI (day after inoculum), 64 (6^th^ of May 2019) to DAI 225 (14^th^ of October 2019), the evaluation of the disease was carried out for 6 times. A visual index for the formed knot was attributed to each wound in each assessment date (value 0 = absence of knot, value 1 = the presence of knot was doubtful, value 2 = visible knot, value 3 = big and well-defined knot), (Fig 2).

For each assessment, the percentage of the sum of visual index 3 and 4 of each plant was used to calculate the Area Under Incidence Progress Curve (AUIPC) index with the following formula:

$$AUIPC=\sum_{i=1}^{n}\left(\frac{y_{i}+y_{i+1}}{2})(t_{i+1}-t_{i}\right)$$

Where: y is the % of the intensity 3 and 4 of the visual index of knot; t is the day of the assessment (i). In the same assessment dates, transversal and longitudinal diameters and height of each knot were measured to calculate the volume, according to an ovoid-based cylinder. On DAI 225 (14^th^ of October, the last assessment date), all knots were removed from the stem using a sterile surgical scalpel and these were individually weighed.

For those knots whose dimensions did not allow a measurement of their diameter, the volume was obtained by the interpolation of volume/weight (volume = 66.58+1352.55*weight, R square 0.84, P value <0.0001).

The transversal diameter of the stem was measured at each height where the knots were formed, and the TCSA calculated to calculate the volume of knot/TCSA index.

Data are available in the BioStudies database (https://www.ebi.ac.uk/biostudies/) under accession number S-BSST1089.

### Metataxonomic analyses

A metataxonomic approach was applied to analyze the total DNA of fourteen samples collected from eleven cultivars of olive plants (R1-R11, Table 2) to study the microbiota composition of the olive knots removed from the stem on DAI 225. Samples R11, R11A, R11B and R11C were collected from the same cultivar (Cultivar Rosciola colli Esini), but from different plants.

**Table 2:** Samples identification (ID) and the relative cultivars of the sampling collection.

Sample-ID	Cultivar
**R1**	cultivar ‘Arbequina’
**R2**	cultivar ‘Ascolana dura’
**R3**	cultivar ‘Ascolana tenera’
**R4**	cultivar ‘Carboncella’
**R5**	cultivar ‘Frantoio’
**R6**	cultivar ‘Fs17®’
**R7**	cultivar ‘Maurino A’
**R8**	cultivar ‘Piantone di Mogliano A’
**R9**	cultivar ‘Piantone di Mogliano B’
**R10**	cultivar ‘Piantone di Falerone’
**R11**	cultivar ‘Rosciola Colli Esini’
**R11A**	cultivar ‘Rosciola Colli Esini’ plant 1
**R11B**	cultivar ‘Rosciola Colli Esini’ plant 2
**R11C**	cultivar ‘Rosciola Colli Esini ‘plant 3

The V3-V4 regions of the 16S rRNA gene were amplified following the parameters defined by Maoloni et al. [26]. Illumina guidelines were used to purify, tag, pool and sequence PCR products. 250-bp paired-end reads were generated by a MiSeq platform (Illumina). Raw files (.fastq) were elaborated by QIIME 2 software [27]. Cutapter was used to remove primer sequences and DADA2 algorithms were employed to denoise the reads [28] through the q2-dada2 plugin in QIIME 2 in order to obtain the ASVs. Taxonomic assignment was performed by the QIIME2 feature-classifier against the Greengenes 16S rRNA gene database v. 13.8. To increase the confidence of sequence reads we excluded the ASVs with less than five read counts in at least two samples. Sequence reads were normalized by verification with an equal representation of 10,000 sequences per sample at the lowest sequence/sample reads. Alpha and beta diversity analyses were performed by QIIME2 diversity plugin. The 16S rRNA gene sequences are available at the Sequence Read Archive of NCBI (accession number PRJNA972624).

### Statistical analysis

One-way ANOVA was performed and, in presence of significant differences, the Tukey-Kramer (HSD) test at P<0.05 was used to detect statistically significant differences among the mean scores. All statistical analyses were performed with JMP 8.0 software (SAS Institute Inc., Cary, NC, USA, 2009).

Differences between alpha or beta diversities and ASVs abundance were analyzed by R software or QIIME2 as appropriate.

## Results

For the identification of the 30 Gram negative isolates collected from olive knots a threshold of 98.65% sequence similarity [29] was set; based on the alignment of the 16S rRNA gene sequences the 30 isolates were ascribed to the following species: *Pseudomonas caspiana*, *Pseudomonas coleopterorum*, *Pseudomonas graminis*, *Pseudomonas lutea*, *Pseudomonas savastanoi*, *Pseudomonas harudinis*, and *Pseudomonas bohemica* (Table 3).

**Table 3:** Identification of the Gram-negative bacteria isolated from olive knots in the University farm “Pasquale Rosati” and a private orchard located at Monte San Vito (AN, Italy).

Field of isolation	Isolate code	Species	Sequence similarity %^a^	E value	Accession N.^b^	Accession N.^c^
Experimental Farm “Pasquale Rosati”	AGU1	*Pseudomonas caspiana*	98.93	0.0	OQ990727	NR_152639.1
	AGU2	*Pseudomonas coleopterorum*	98.89	0.0	OQ990728	NR_137215.1
	AGU3	*Pseudomonas caspiana*	99.11	0.0	OQ990729	NR_152639.1
	AGU4	*Pseudomonas graminis*	99.41	0.0	OQ990730	NR_026395.1
	AGU6	*Pseudomonas lutea*	99.22	0.0	OQ990731	NR_029103.1
	AGU8	*Pseudomonas graminis*	99.63	0.0	OQ990732	NR_026395.1
	AGU9	*Pseudomonas caspiana*	99.72	0.0	OQ990733	NR_152639.1
	AGU10	*Pseudomonas caspiana*	99.53	0.0	OQ990734	NR_152639.1
	AGU11	*Pseudomonas caspiana*	99.47	0.0	OQ990735	NR_152639.1
	AGU13	*Pseudomonas graminis*	99.51	0.0	OQ990736	NR_026395.1
	AGU14	*Pseudomonas caspiana*	99.33	0.0	OQ990737	NR_152639.1
	AGU15	*Pseudomonas graminis*	99.80	0.0	OQ990738	NR_026395.1
	AGU17	*Pseudomonas graminis*	99.30	0.0	OQ990739	NR_026395.1
	AGU18	*Pseudomonas lutea*	99.12	0.0	OQ990740	NR_029103.1
	AGU19	*Pseudomonas caspiana*	99.35	0.0	OQ990741	NR_152639.1
	AGU20	*Pseudomonas caspiana*	99.22	0.0	OQ990742	NR_152639.1
	AGU21	*Pseudomonas graminis*	99.49	0.0	OQ990743	NR_026395.1
Orchard located at Monte San Vito	SAN1	*Pseudomonas savastanoi*	99.81	0.0	OQ990744	NR_117822.1
	SAN6	*Pseudomonas caspiana*	99.43	0.0	OQ990745	NR_152639.1
	SAN8	*Pseudomonas caspiana*	99.48	0.0	OQ990746	NR_152639.1
	SAN11	*Pseudomonas caspiana*	99.61	0.0	OQ990747	NR_152639.1
	SAN12	*Pseudomonas bohemica*	99.15	0.0	OQ990748	NR_159101.1
	SAN13	*Pseudomonas harudinis*	98.81	0.0	OQ990749	NR_181730.1
	SAN15	*Pseudomonas harudinis*	98.73	0.0	OQ990750	NR_181730.1
	SAN16	*Pseudomonas bohemica*	98.71	0.0	OQ990751	NR_159101.1
	SAN18	*Pseudomonas caspiana*	99.66	0.0	OQ990752	NR_152639.1
	SAN19	*Pseudomonas harudinis*	98.80	0.0	OQ990753	NR_181730.1
	SAN20	*Pseudomonas harudinis*	98.73	0.0	OQ990754	NR_181730.1
	SAN21	*Pseudomonas caspiana*	99.17	0.0	OQ990755	NR_152639.1
	SAN22	*Pseudomonas caspiana*	99.55	0.0	OQ990756	NR_152639.1

^a^ Similarity % between the analyzed sequence and the GenBank sequence.

^b^ Access number of the analyzed sequence deposited and available at the GenBank database.

^c^ Access number of the GenBank sequence with the highest identity % with the analyzed sequence.

**Fig 3:**
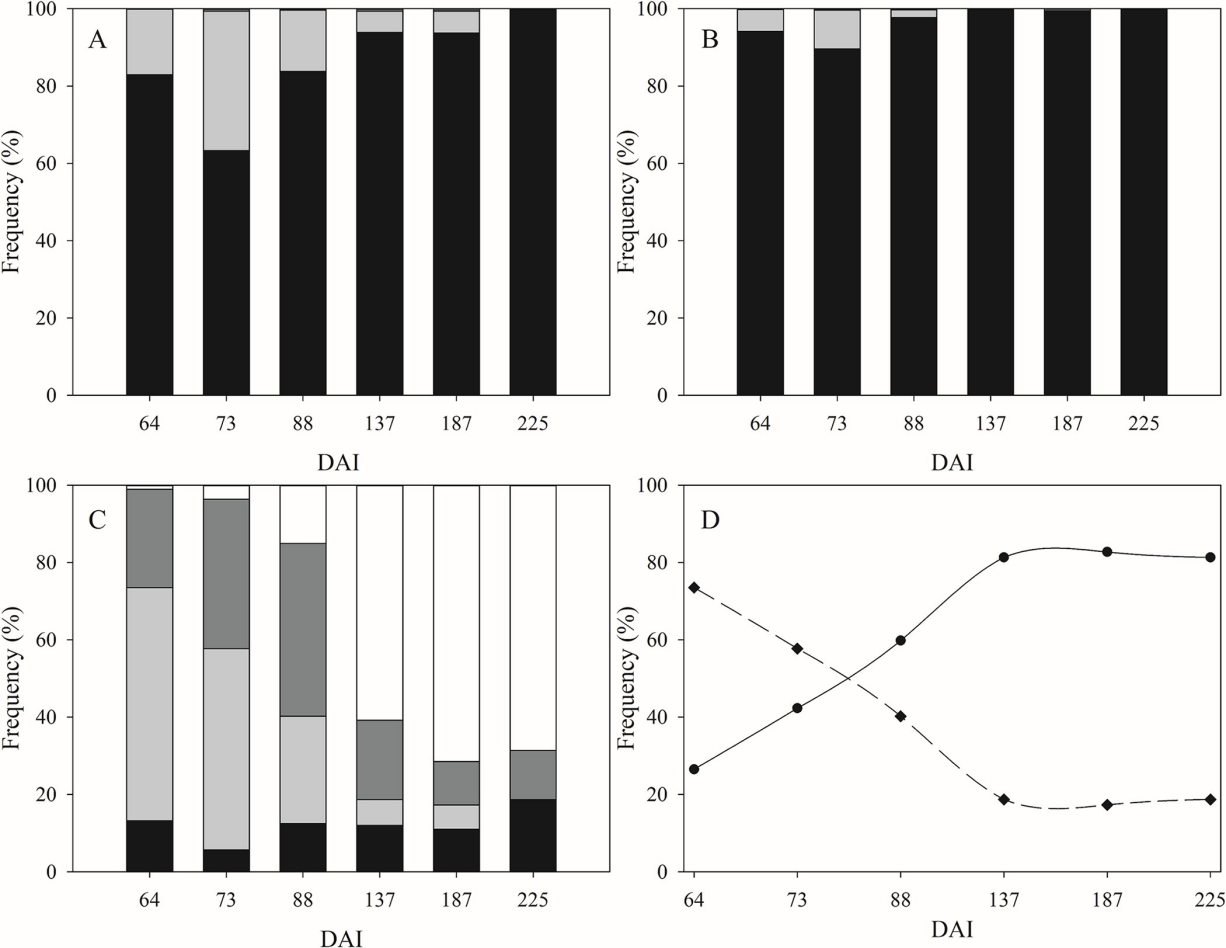
Evolution of the frequency of the visual indexes for the formed knot attributed to each wound in time (DAI: Day after inoculum) on the whole set of plants and cultivar tested. (A) Non-inoculated wounds in the non-inoculated plants (control); (B) Non-inoculated wounds in the upper section in the inoculated plants; (C) and (D) Inoculated wounds in the lower section in the inoculated plants. Graph A, B, and C: value 0 = absence of knot (■), value 1 = the presence of knot was doubtful (■), value 2 = visible knot (■), value 3 = big and well-defined knot (□). Graph D: value 0 plus 1 (slashed line), value 2 plus 3 (continuous line).

The wounds inoculated in the lower section of stems showed a growing trend for the values 2 and 3 in the visual index for the formed knot, opposite to values 0 and 1. The trend is best shown if value 0 is added to value 1, and value 2 is added to value 3 (Fig 3D). In fact, the first couple of values decreased until 18%, whereas the second couple reached the 82% of the total of wounds.

Contrary to the inoculated wounds (on lower section), the non-inoculated wounds (on upper section) limited rates of value 2 and 3 in the visual index for the formed knots. The maximum value as the sum of values 2 and 3 corresponded to about 0.5% of the total non-inoculated wounds, recorded on DAI 187 (September 6^th^, Fig 3B).

In the non-inoculated plants (control), the value 2 of the visual index for the formed knots reached the maximum intensity on DAI 137 (July 18^th^), corresponding to 0.55% of the total of the non-inoculated wounds (Fig 3A). On DAI 187 (September 6^th^) the maximum degree reached for values 2 plus 3, that corresponded to 0.56% of the total of non-inoculated wounds. The value 1 increased from 64 DAI (May 6^th^, 17%) to 73 DAI (May 15^th^, 36%) before it fell down. On 225 DAI (October 14^th^) value 1 was not used.

**Fig 4:**
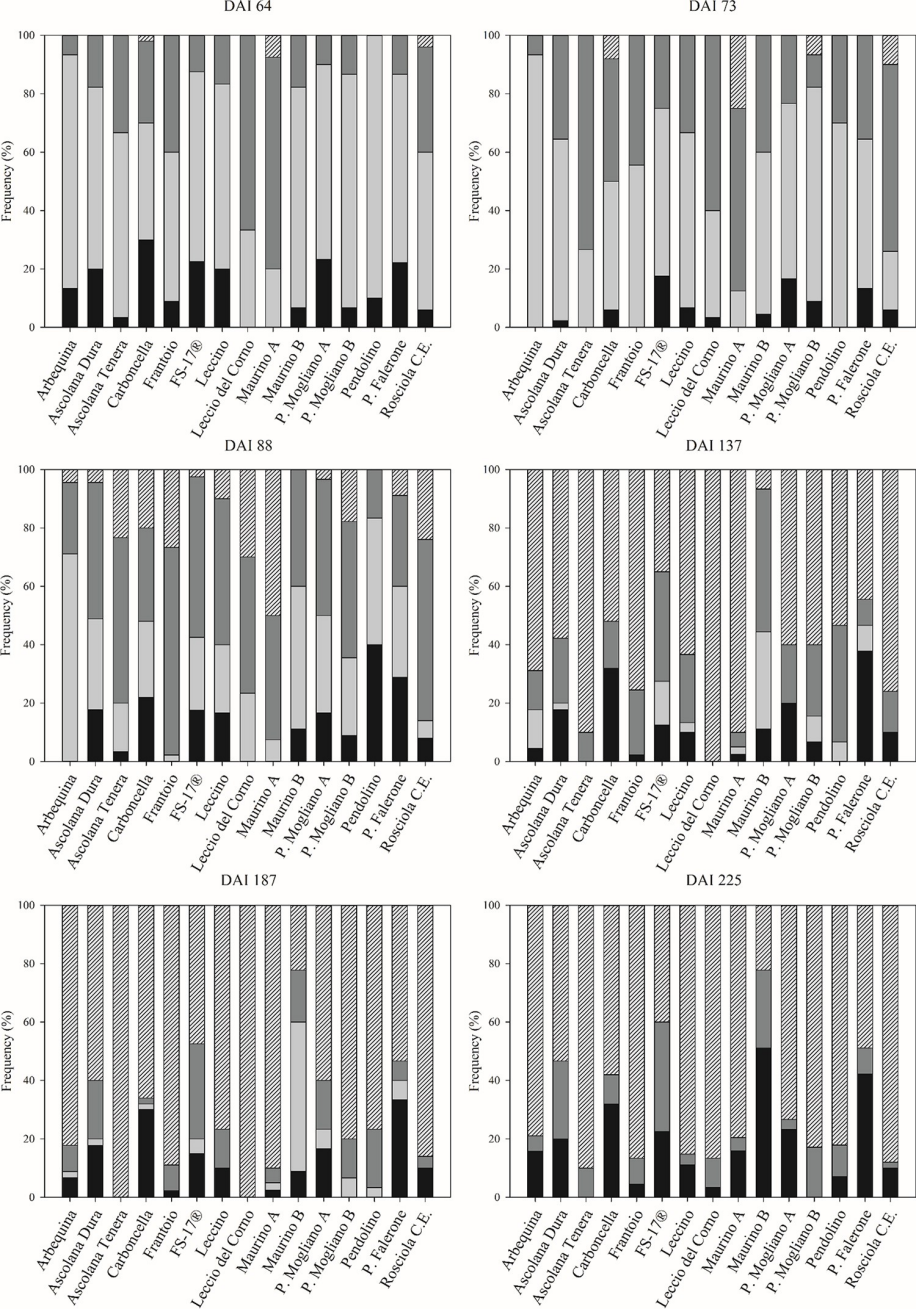
Evolution of the frequency per cultivar of the visual indexes for the formed knot attributed to each inoculated wound (the wounds in the lower section) of each treated plant in time (DAY: Day after inoculum). Value 0 = absence of knot (■), value 1 = the presence of knot was doubtful (■), value 2 = visible knot (■), value 3 = big and well-defined knot (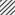).

On DAI 64 (May 6^th^) all cultivars except ‘Pendolino’ formed knots and showed values 2 and 3 of the visual index. Instead, the ‘Pendolino’ cultivar showed the value 2 starting from DAI 73 (May 15^th^, Fig 4).

The severity of the disease followed an increasing trend starting from DAI 64 (May 5^th^). On this day only ‘Carboncella’, ‘Maurino A’ and ‘Rosciola Colli Esini’ cultivar already showed the presence of big tumors (value 3, Fig 4).

On 18^th^ July (137 DAI) 100% of the inoculated wounds formed knots in ‘Leccio del corno’, followed by ‘Maurino A’ and ‘Ascolana Tenera’ with 90.0%, ‘Rosciola colli Esini’ with 76.0%, ‘Frantoio’ with 75.6%, ‘Arbequina’ with 68.9%, ‘Leccino’ with 63.3%, ‘Piantone di Mogliano A’ and ‘Piantone di Mogliano B’ with 60.0%, ‘Ascolana Dura’ with 57.8%, ‘Pendolino’ with 53.3%, ‘Carboncella’ with 52.0%, ‘Piantone di Falerone’ with 44.4%, ‘FS-17®’ with 35.0%, and ‘Maurino B’ with 6.67%.

On 137 DAI all the cultivars reached percentages higher than 50% of knots-forming wounds. In DAI 225 “Maurino B” and ‘Piantone di Falerone’ cultivars presented the highest percentage of absence of knot (value 0 of the visual index), showing 51% and 42% of the inoculated wounds, respectively. In the same DAI 225 ‘Piantone di Mogliano A’ showed 23% of the inoculated wounds without knots, while ‘Piantone di Mogliano B’ cultivar showed the presence of knot in all of the inoculated wounds. ‘Maurino B’ showed approximately the 51% of the inoculated wounds without knots, which corresponds to an increase of 35% compared to ‘Maurino A’ (16%). ‘Maurino A’ had also the highest AUIPC value, while ‘Maurino B’ showed the lower (149 and 73 respectively).

**Fig 5:**
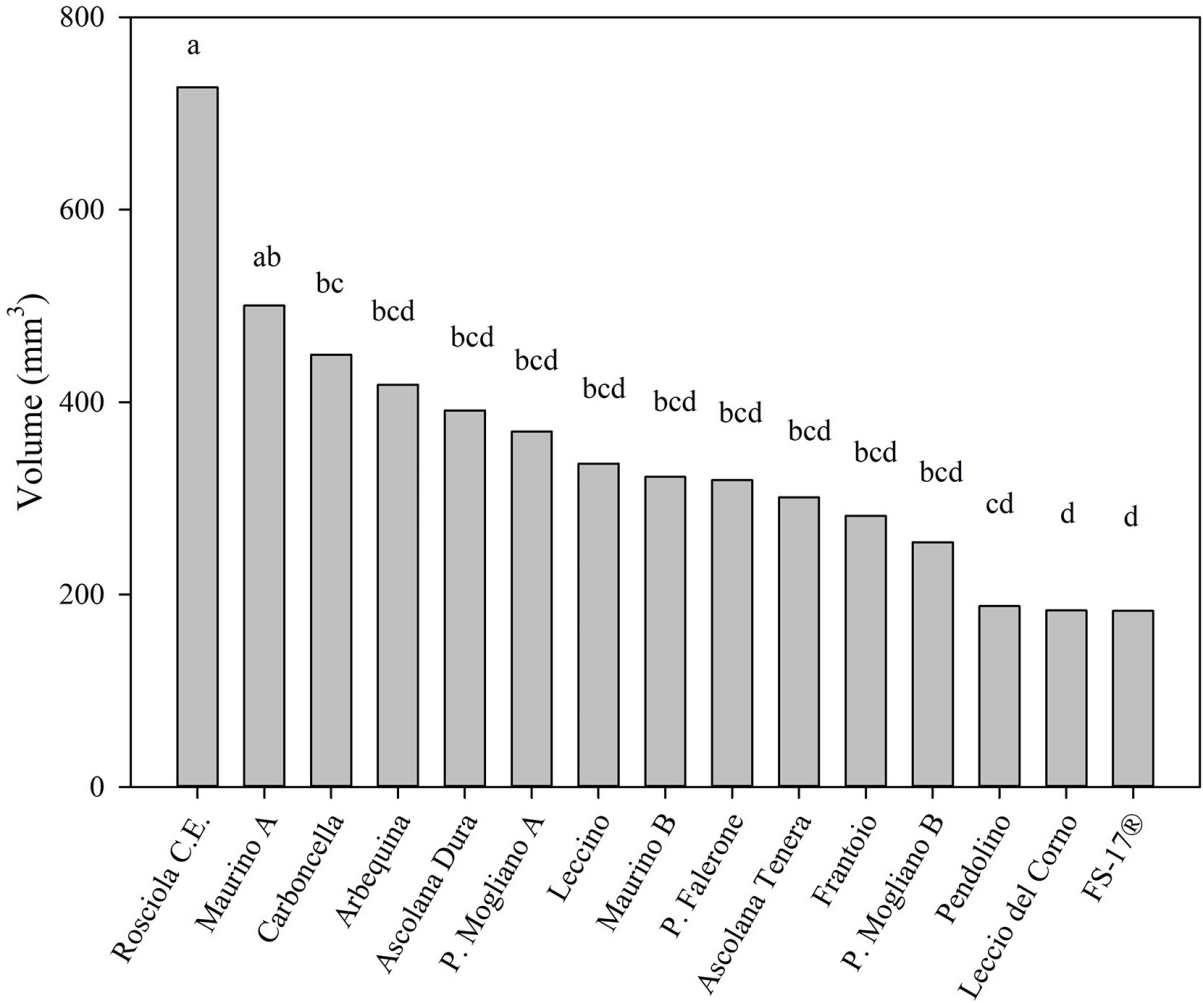
Mean in each cultivar of the volume of the knots at the value 2 and 3 of the visual index at 225 DAI. Each bar represents the mean value of a different number of replicates: ‘Rosciola C.E.’, 44; ‘Maurino A’, 33; ‘Carboncella’, 34; ‘Arbequina’, 31; ‘Ascolana Dura’, 29; ‘P. Mogliano A’, 23; ‘Leccino’, 19; ‘Maurino B’, 9; ‘P. Falerone’, 22; ‘Ascolana Tenera’, 28; ‘Frantoio’, 41; ‘P. Mogliano B’, 20; ‘Pendolino’, 22; ‘Leccio del Corno’, 29; ‘FS-17^®^’, 26. Different letters indicate significant differences among cultivars according to the Tukey test (α = 0.05), P.value: <0.0001.

Considering the values 2 and 3 of the visual index of the presence of the knot, ‘Rosciola Colli Esini’ showed the highest mean value of knot volume (727±511 mm^3^, Fig 5). Only ‘Maurino A’ presented a significantly comparable knot (494±335 mm^3^) to ‘Rosciola Colli Esini’, and a slight, but not significant, difference with ‘Maurino B’ (322±104 mm^3^). Also ‘Piantone di Mogliano A’ and ‘Piantone di Mogliano B’ did not present significantly difference on knot volumes (369±188 mm^3^ and 254±190 mm^3^).

Although ‘Leccio del Corno’ showed the greater intensity of the disease than other cultivars (Fig 4), the severity of the disease was low (164 ± 72 mm^3^), although it showed significant differences only with ‘Rosciola Colli Esini’, ‘Maurino A’, and ‘Carboncella’ (Fig 5).

**Fig 6:**
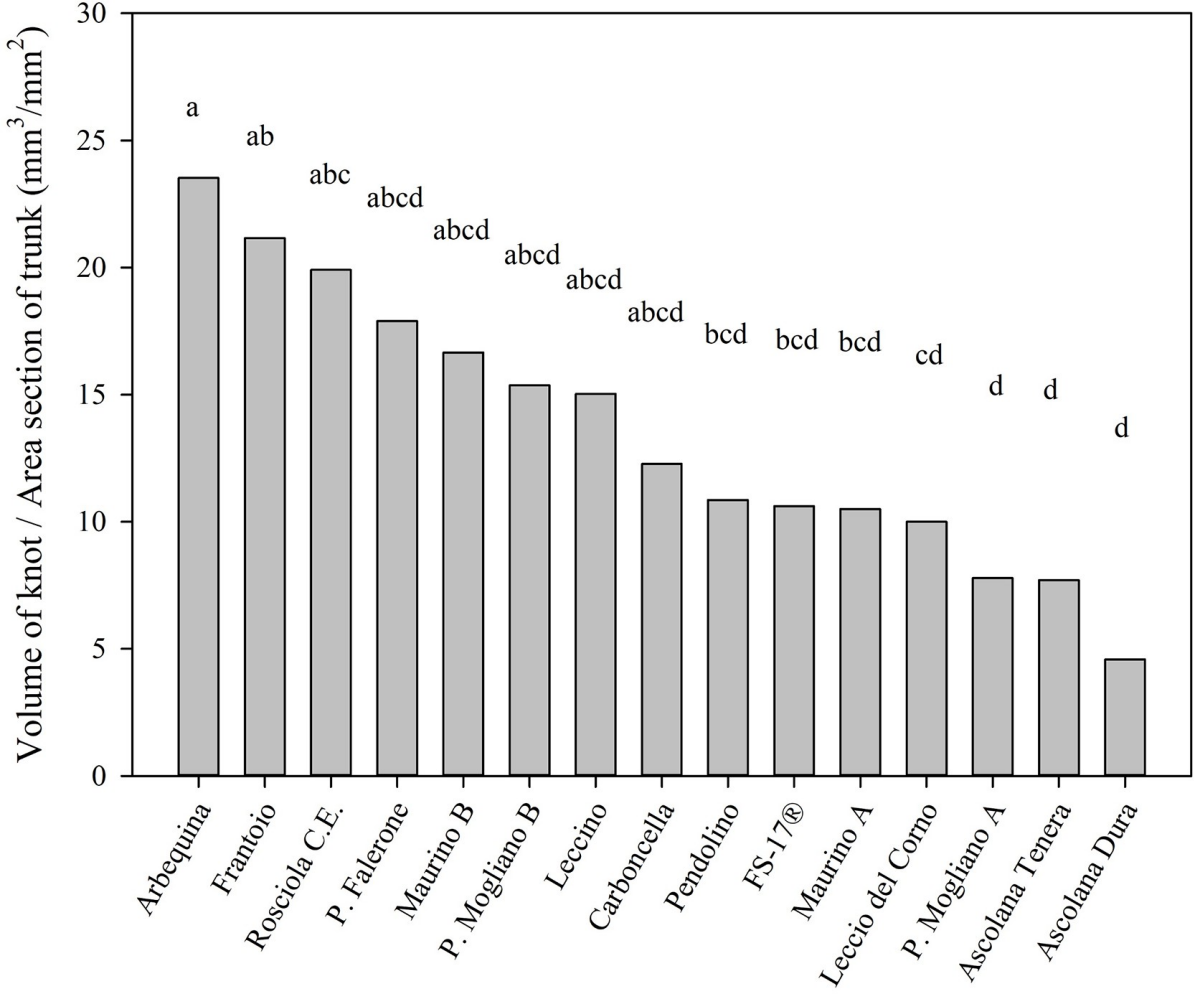
Relation between tumor volume and the area of the stem section where the knot was positioned at DAI 225. Each bar represents the mean value of a different number of replicates: ‘Arbequina’, 23; ‘Frantoio’, 32; ‘Rosciola C.E.’, 40; ‘P. Falerone’, 20; ‘Maurino B’, 9; ‘P. Mogliano B’, 20; ‘Leccino’, 9; ‘Carboncella’, 24; ‘Pendolino’, 21; ‘FS-17^®^’, 21; ‘Maurino A’, 25; ‘Leccio del Corno’, 29; ‘P. Mogliano A’, 23; ‘Ascolana Tenera’, 18; ‘Ascolana Dura’, 17. Different letters indicate significant differences among cultivars according to the Tukey test (α = 0.05), P.value: <0.0001.

Regarding the index ‘volume of knot / TCSA’, ‘Maurino A’ and ‘Maurino B’, ‘Piantone di Mogliano A’ and ‘Piantone di Mogliano B’ showed similar values without significant differences (Fig 6).

AUIPC of each cultivar: ‘Arbequina’ 107, ‘Ascolana Dura’ 111, ‘Ascolana tenera’ 148, ‘Carboncella’ 100, ‘Frantoio’ 148, ‘FS-17’ 108, ‘Leccino’ 123, ‘Leccio del Corno’ 147, ‘Maurino A’ 149, ‘Maurino B’ 73, ‘Piantone di Mogliano A’ 107, ‘Piantone di Mogliano B’ 125, ‘Pendolino 115’, ‘Piantone di Falerone’ 81, ‘Rosciola’ 139.

**Fig 7:**
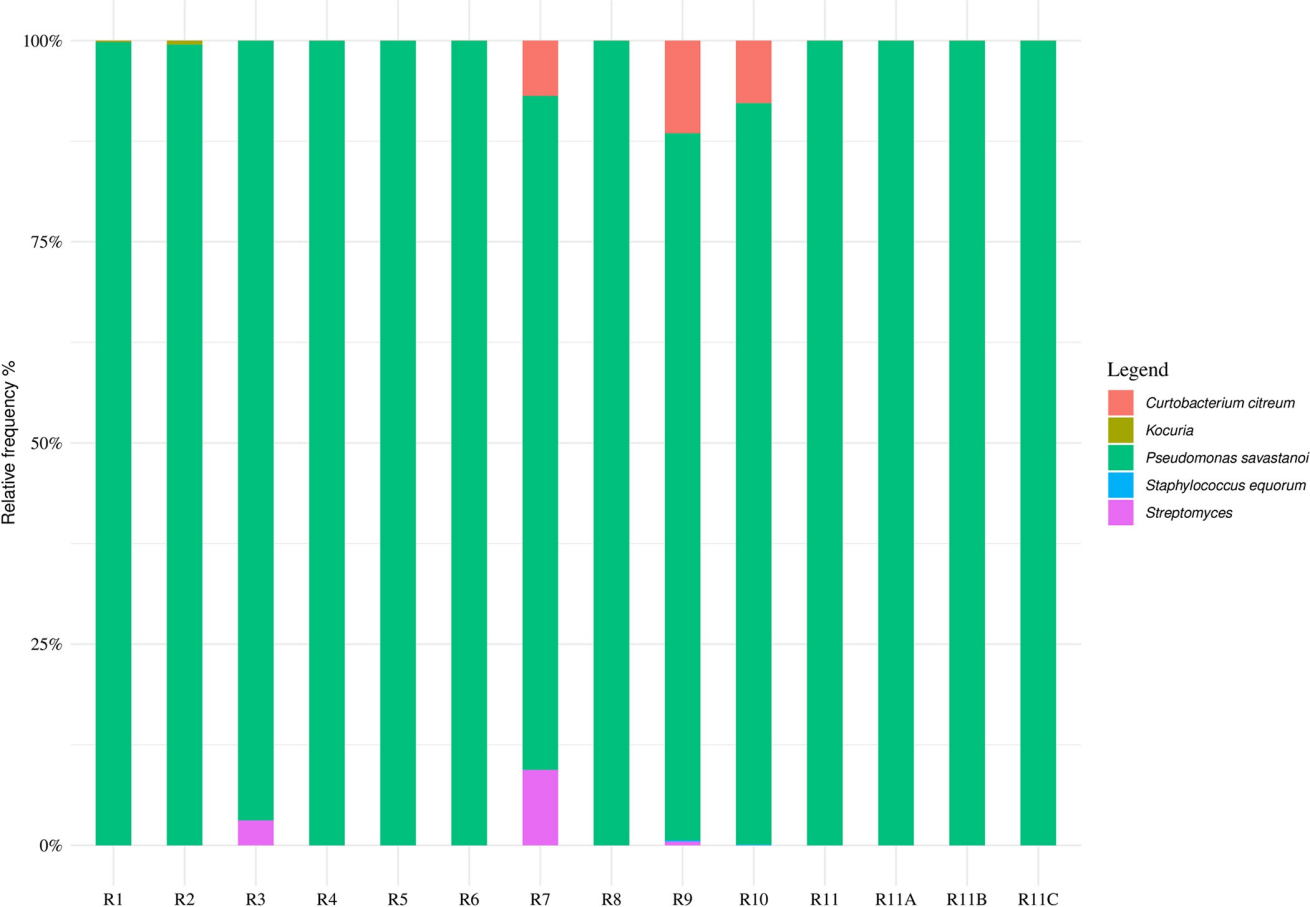
Relative frequency of bacterial Amplicon Sequencing Variants (ASVs) detected in the olive knots removed from the stem of different cultivars on DAI (day after inoculation) 225. For the coding of the samples see Table 2.

Regarding the metataxonomic composition of olive knots removed from the stem of different cultivars on DAI 225, no significant differences in alpha or beta-diversity indexes were found (S1 Table, S1 Fig). Comparison of ASVs relative abundances between the cultivars confirmed the dominance of Pss. Its relative frequency was never less than 80%. Pss represented the only identified taxon in the knots collected from cultivar ‘Carboncella’ (R4), ‘Frantoio’ (R5), Fs17 (R6), ‘Piantone di Mogliano A’ (R8) and ‘Rosciola Colli Esini’ (R11) (Fig 7).

Additional species were also detected at low relative abundances in knots of some cultivars. More specifically, ‘Arbequina’ (R1) and ‘Ascolana dura’ (R2) showed the presence of *Kocuria* in percentage less than 0.5% of the relative frequency (Fig 7), whereas *Streptomyces* was found in knots sampled from cultivar ‘Ascolana tenera’ (R3–3% of the relative frequency), ‘Maurino A’ (R7–9.3% of the relative frequency) and ‘Piantone di Mogliano B’ (R9–0.5% of the relative frequency) (Fig 7). The cultivar ‘Maurino A’ (R7) showed also the presence of *Curtobacterium citreum* (6.8% of the relative frequency). This taxon was found also in knots collected from cultivar ‘Piantone di Mogliano B’ (R9) and ‘Piantone di Falerone’ (R10) together with *Staphylococcus equorum* (~ 0.11% of the relative frequency) (Fig 7).

By comparing the results collected from different plants of the cultivar ‘Rosciola Colli Esini’, this latter was characterized by the exclusive presence of *Pseudomonas savastanoi* (100% of the relative frequency) (Fig 7).

## Discussion

All the wounds inoculated with the pathobiome consortium of *Pseudomonas* species showed a high percentage of disease. On the contrary, the wounds in the not inoculated control plants did not show the disease. This evidence clearly suggests that the bacterial inoculum was responsible for the infection of the treated olive trees. This evidence was confirmed also by the metataxonomic approach analysis, which evidenced that *Pseudomonas savastanoi* pv *savastanoi* was more present than other species, in all the cultivars. Contrary to what was reported by Penyalver et al. [1], the absence of knots in non-inoculated wounds of treated plants indicates that bacteria of the consortium were unable to transmigrate within the tested time. This latter finding suggests that the spread of the disease can be confined to the inoculated wounds if the environmental condition are suitable and compartmentalization and/or a prompt disinfection of the trees is executed.

Furthermore, the cultivars herein treated differed in the time of appearance of knot even though after about 187 days since the inoculation, wounds reached the maximum percentage of knots (2 and 3). In the field the time of appearance of the disease is important to plan for appropriate disinfection treatments.

Moreover, the fact that all cultivars with the only exception of ‘Maurino B’ showed a high percentage of wounds with knots (more than 50%), indicates that all assayed varieties are susceptible to *Pseudomonas savastanoi* pv. *savastanoi*. As already reported by Penyalver et al. [1], plants’ age might affect the incidence of this disease, with younger plants showing lower levels of olive knot. This is in alignment with the results we found for ‘Maurino’ where younger plants showed higher levels of intensity of disease and the AUIPC but not valid for ‘Piantone di Mogliano’, where higher levels of disease were found in older plants. Such different behavior legitimizes the need for conducting further research with emphasis about the susceptibility to *Pseudomonas* according to the age or the physiological status of olive shoots and plants.

Valverde et al. [17] showed how after a late frost ‘Rosciola Colli Esini’ was into the “low susceptibility” cluster, with low level of disease and low level of injury due to frost (confirmed also by Lodolini e al. [30, 31]). In the present investigation, opposite results to those reported by these authors were obtained in protected environment with young pot trees. In fact, after inoculation with the bacterial consortium, ‘Rosciola Colli Esini’ was the most susceptible cultivar among those assayed.

Hence, we can hypothesize that the formation of wounds on olive tree tissues might be the first step to trigger the disease; the higher tolerance of this cultivar to late frost can be determined by the lower presence of damages due to frost. Consequently, a modest presence of the disease is not due to a lower sensibility of the plant to the inoculum.

Moreover, the dominance of *Pseudomonas savastanoi* in the knots sampled on DAI 225 is congruent with the available literature, describing this microorganism as the primary causative agent of olive knot disease [2, 17, 32]. By contrast, the genera *Kocuria* and *Streptomyces*, and the species *Curtobacterium citreum* and *Staphylococcus equorum* detected to a lesser extent in some cultivars might likely be part of the resident epiphytic and endophytic microflora of olive trees, as previously suggested [33, 34]. The absence of the other *Pseudomonas* species besides *Pseudomonas savastanoi* indicates that they didn’t develop in the knot after inoculation.

## Conclusion

The present study confirms that all cultivars herein assayed are susceptible to olive knot, while the reaction time, intensity, and severity of the disease depend on the olive cultivar. This information is supportive of design decisions for new olive groves in less endemic areas with well adapted varieties to limit the risk of frost damages in the shoots and stems and then the severity of the knot disease. Furthermore, all the information herein collected and the observed inability of the inoculated bacteria to transmigrate in the tested time might help the olive grower to reduce the spread of the disease in the field by pruning the infected portion of the trees. Maintaining only the healthy parts of the branches is a priority for orchard sanitation, but it is not mandatory because from the knots the Pss does not migrate from a wound to other wounds along the same stem. There is the need for studies about other bacterial populations to better understand the interaction of the Pss with other bacterial species, after natural and artificial inoculation and possibly to improve the use of biological treatments in the olive groves.

## Supporting information

S1 Table Alpha diversity measurement for 16S rRNA amplicons analyzed. (PDF)

S1 Fig Principal coordinates analysis of Bray Curtis distances for 16S rRNA gene sequence data. Samples are color-coded by variety.
(PDF)

## References

[1] Penyalver R., García A., Ferrer A., Bertolini E., Quesada J.M., Salcedo C.I., et al. Factors Affecting *Pseudomonas savastanoi* pv. *savastanoi* Plant Inoculations and Their Use for Evaluation of Olive Cultivar Susceptibility. Phytopathology 2006 Mar; 96(3):313–9. doi: 10.1094/PHYTO-96-0313 . 18944447

[2] Quesada J. M., Penyalver R., Pérez‐Panadés J., Salcedo C. I., Carbonell E. A., & López M. M. Dissemination of *Pseudomonas savastanoi* pv. *savastanoi* populations and subsequent appearance of olive knot disease. Plant Pathology 2010, 59(2), 262–269.

[3] Wilson E. E. The olive knot disease: Its inception, development and control. Hilgardia 1935, 9:231–264.

[4] Gardan L., Bollet C., Abu Ghorrah M., Grimont F., Grimont P. A. D. DNA relatedness among the pathovar strains of *Pseudomonas syringae* subsp. *savastanoi* Janse (1982) and proposal of *Pseudomonas savastanoi* sp. nov. Int. J. 1992.

[5] Bouaichi A., Benkirane R., El-Kinany S., Habbadi K., Lougraimzi H., Sadik S., et al. Potential effect of antagonistic bacteria in the management of olive knot disease caused by *Pseudomonas savastanoi* pv. *savastanoi*. *Journal of Microbiology*, *Biotechnology and Food Sciences* 2019, 8(4), 1035–1040. 10.15414/jmbfs.2019.8.4.1035-1040

[6] Buonaurio R., Moretti C., Da Silva D. P., Cortese C., Ramos C., Venturi V. The olive knot disease as a model to study the role of interspecies bacterial communities in plant disease. *Frontiers in Plant Science* 2015, 6 (June), 1–12. 10.3389/fpls.2015.00434 26113855 PMC4461811

[7] da Silva D. P., Castañeda-Ojeda M. P., Moretti C., Buonaurio R., Ramos C., & Venturi V. Bacterial multispecies studies and microbiome analysis of a plant disease. *Microbiology (United Kingdom)* 2014, 160 (PART 3), 556–566. 10.1099/mic.0.074468-0 24421406

[8] Fernandes A., & Marcelo M. A possible synergistic effect of *Erwinia* sp. on the development of Olive knot symptoms caused by *pseudomonas syringae* pv *savastanoi* in *Olea europaea*. *Acta Horticulturae* 2002, 586, 729–731. 10.17660/ActaHortic.2002.586.156

[9] Marchi G., Sisto A., Cimmino A., Andolfi A., Cipriani M. G., Evidente A., et al. Interaction between *Pseudomonas savastanoi* pv. *savastanoi* and *Pantoea agglomerans* in olive knots. *Plant Pathology* 2006, 55(5), 614–624. 10.1111/j.1365-3059.2006.01449.

[10] Mirik M., Aysan Y. E. Ş. İ. M., & Sahin F. Characterization of *Pseudomonas savastanoi* pv. *savastanoi* strains isolated from several host plants in Turkey and report of fontanesia as a new host. Journal of Plant Pathology 2011, 263–270.

[11] Azadam H.R., Cooksey D.A. A semiselective medium for detecting ephiphytic and systemic populations of *Pseudomonas savastanoi* from Oleander. Phytopathology 1995, 85, 740–745.

[12] Bozkurt I. A., Soylu S., Mirik M., Ulubas Serce C., & Baysal Ö. Characterization of bacterial knot disease caused by P seudomonas savastanoi pv. savastanoi on pomegranate (Punica granatum L.) trees: a new host of the pathogen. Letters in applied microbiology 2014, 59(5), 520–527. doi: 10.1111/lam.12309 25039423

[13] Schiff S., Tani C., Cimmino A., Mandala G., Cinelli T., Evidente A., et al. The colonization processes of Myrtus communis by strains of *Pseudomonas savastanoi* with a differential ability to produce phytohormones. Plant Pathology 2019, 68(6), 1109–1119.

[14] Hosni T., Moretti C., Devescovi G., Suarez-Moreno Z. R., Fatmi M. B., Guarnaccia C., et al. Sharing of quorum-sensing signals and role of interspecies communities in a bacterial plant disease. *ISME Journal* 2011, 5(12), 1857–1870. doi: 10.1038/ismej.2011.65 21677694 PMC3223305

[15] Mina D., Pereira J. A., Lino-Neto T., & Baptista P. Impact of plant genotype and plant habitat in shaping bacterial pathobiome: a comparative study in olive tree. *Scientific Reports* 2020, 10(1), 3475. doi: 10.1038/s41598-020-60596-0 32103149 PMC7044170

[16] Sisto A., Cipriani M. G., Morea M. Knot formation caused by *Pseudomonas syringae* subsp. *savastanoi* on olive plants is hrp-dependent. *Phytopathology* 2004, 94(5), 484–489. 10.1094/PHYTO.2004.94.5.484 18943767

[17] Valverde P., Zucchini M., Polverigiani S., Lodolini E. M., López-Escudero F. J., & Neri D. Olive knot damages in ten olive cultivars after late-winter frost in central Italy. *Scientia Horticulturae* 2020, 266 (October 2019), 109274. 10.1016/j.scienta.2020.109274

[18] Varvaro L., Surico G. Comportamento di diverse cultivars di Olivo (Olea europaea L.) alla inoculazione artificiale con *Pseudomonas savastanoi* (EF Smith) Stevens. Phytopathologia mediterranea 1978, 174–177.

[19] Teviotdale B. L., Krueger W. H. Effects of timing of copper sprays, defoliation, rainfall, and inoculum concentration on incidence of olive knot disease. *Plant Disease* 2004, 88(2), 131–135. doi: 10.1094/PDIS.2004.88.2.131 30812418

[20] Benjama A. Étude de la sensibilité variétale de l’olivier au Maroc vis-à-vis de *Pseudomonas syringae* pv. savastanoi, agent de la tubercolose. Cahiers Agric. 1994, 3 (6), 405–408.

[21] Salman M., Greenhut R., Preece J., Ferguson L., Kluepfel D. Field evaluation of olive (Olea europaea) genotypes for resistance to *Pseudomonas savastanoi* pv. *savastanoi*. J Plant Pathol 2020, 102, 663–670 (2020). 10.1007/s42161-020-00549-8

[22] Hynes W. L., Ferretti J. J., Gilmore M. S., & Segarra R. A. PCR amplification of streptococcal DNA using crude cell lysates. FEMS microbiology letters 1992, 94(1–2), 139–142. doi: 10.1016/0378-1097(92)90597-h 1521762

[23] Osimani A., Garofalo C., Aquilanti L., Milanović V., & Clementi F. Unpasteurised commercial boza as a source of microbial diversity. International Journal of Food Microbiology 2015, 194, 62–70. doi: 10.1016/j.ijfoodmicro.2014.11.011 25437059

[24] Weisburg W. G., Barns S. M., Pelletier D. A., & Lane D. J. 16S ribosomal DNA amplification for phylogenetic study. *Journal of bacteriology* 1991, 173(2), 697–703. doi: 10.1128/jb.173.2.697-703.1991 1987160 PMC207061

[25] Altschul S. F., Gish W., Miller W., Myers E. W., & Lipman D. J. Basic local alignment search tool. Journal of molecular biology 1990, 215(3), 403–410. doi: 10.1016/S0022-2836(05)80360-2 2231712

[26] Maoloni A., Ferrocino I., Milanović V., Cocolin L., Corvaglia M. R., Ottaviani D., et al. The microbial diversity of non-Korean kimchi as revealed by viable counting and metataxonomic sequencing. Foods 2020, 9, 1568. doi: 10.3390/foods9111568 33137924 PMC7693646

[27] Bolyen E., Rideout J. R., Dillon M. R., Bokulich N. A., Abnet C. C., Al-Ghalith G. A., et al. Reproducible, interactive, scalable and extensible microbiome data science using QIIME 2. Nature Biotechnology 2019, 37(8), 852–857. doi: 10.1038/s41587-019-0209-9 31341288 PMC7015180

[28] Callahan B. J., McMurdie P. J., Rosen M. J., Han A. W., Johnson A. J. A., & Holmes S. P. DADA2: High-resolution sample inference from Illumina amplicon data. Nature Methods 2016, 13(7), 581–583. doi: 10.1038/nmeth.3869 27214047 PMC4927377

[29] Kim M., Oh H. S., Park S. C., & Chun J. Towards a taxonomic coherence between average nucleotide identity and 16S rRNA gene sequence similarity for species demarcation of prokaryotes. International journal of systematic and evolutionary microbiology 2014, 64 (Pt_2), 346–351. doi: 10.1099/ijs.0.059774-0 24505072

[30] Lodolini E. M., Alfei B., Santinelli A., Cioccolanti T., Polverigiani S., & Neri D. Frost tolerance of 24 olive cultivars and subsequent vegetative re-sprouting as indication of recovery ability. *Scientia Horticulturae* 2016, 211, 152–157. 10.1016/j.scienta.2016.08.025

[31] Lodolini E.M., Alfei B., Cioccolanti T., Zucchini M. and Neri D. Comparison of frost damages in eleven olive cultivars after two freezing events in central Italy. Acta Hortic 2022. 1346, 161–168 doi: 10.17660/ActaHortic.2022.1346.21

[32] Rodríguez‐Moreno L., Jiménez A. J., & Ramos C. Endopathogenic lifestyle of Pseudomonas savastanoi pv. savastanoi in olive knots. Microbial Biotechnology 2009, 2(4), 476–488. doi: 10.1111/j.1751-7915.2009.00101.x 21255279 PMC3815908

[33] Filiz Doksöz S., & Bozkurt I. A. Biological control of Pseudomonas savastanoi pv. savastanoi causing the olive knot disease with epiphytic and endophytic bacteria. Journal of Plant Pathology 2022, 104(1), 65–78.

[34] Mina D., Pereira J. A., Lino-Neto T., & Baptista P. Epiphytic and endophytic bacteria on olive tree phyllosphere: exploring tissue and cultivar effect. Microbial ecology 2020, 80(1), 145–157. doi: 10.1007/s00248-020-01488-8 31965223

