# Supplementary material for: Knot formation and spread along the shoot stem in 13 olive cultivars inoculated with an indigenous pathobiome of 7 species of Pseudomonas including Pseudomonas savastanoi
Source: PLoS One. 2023 Aug 11;18(8):e0289875. doi: 10.1371/journal.pone.0289875 (PMC10420344; doi:10.1371/journal.pone.0289875)
Supplement: S1 Table — (PDF) [file pone.0289875.s001.pdf]

S1 Table. Alpha diversity measurement for 16S rRNA amplicons analyzed.

Sample code	Chao1	Observation	Shannon
**R10**	231.10	219	6.569
**R11**	74.00	74	5.729
**R11A**	84.20	83	5.828
**R11B**	81.00	80	5.737
**R11C**	101.75	98	5.849
**R3**	81.00	81	5.930
**R4**	98.00	86	5.871
**R5**	79.00	79	5.695
**R6**	76.00	76	5.777
**R7**	391.38	387	7.418
**R8**	95.50	95	5.857
**R9**	228.00	216	6.147
**R1**	100.19	100	5.812
**R2**	94.75	91	5.745
