# Supplementary material for: Knot formation and spread along the shoot stem in 13 olive cultivars inoculated with an indigenous pathobiome of 7 species of Pseudomonas including Pseudomonas savastanoi
Source: PLoS One. 2023 Aug 11;18(8):e0289875. doi: 10.1371/journal.pone.0289875 (PMC10420344; doi:10.1371/journal.pone.0289875)

S1 Fig. Principal coordinates analysis of Bray Curtis distances for 16S rRNA gene sequence data. Samples are color-coded by variety.

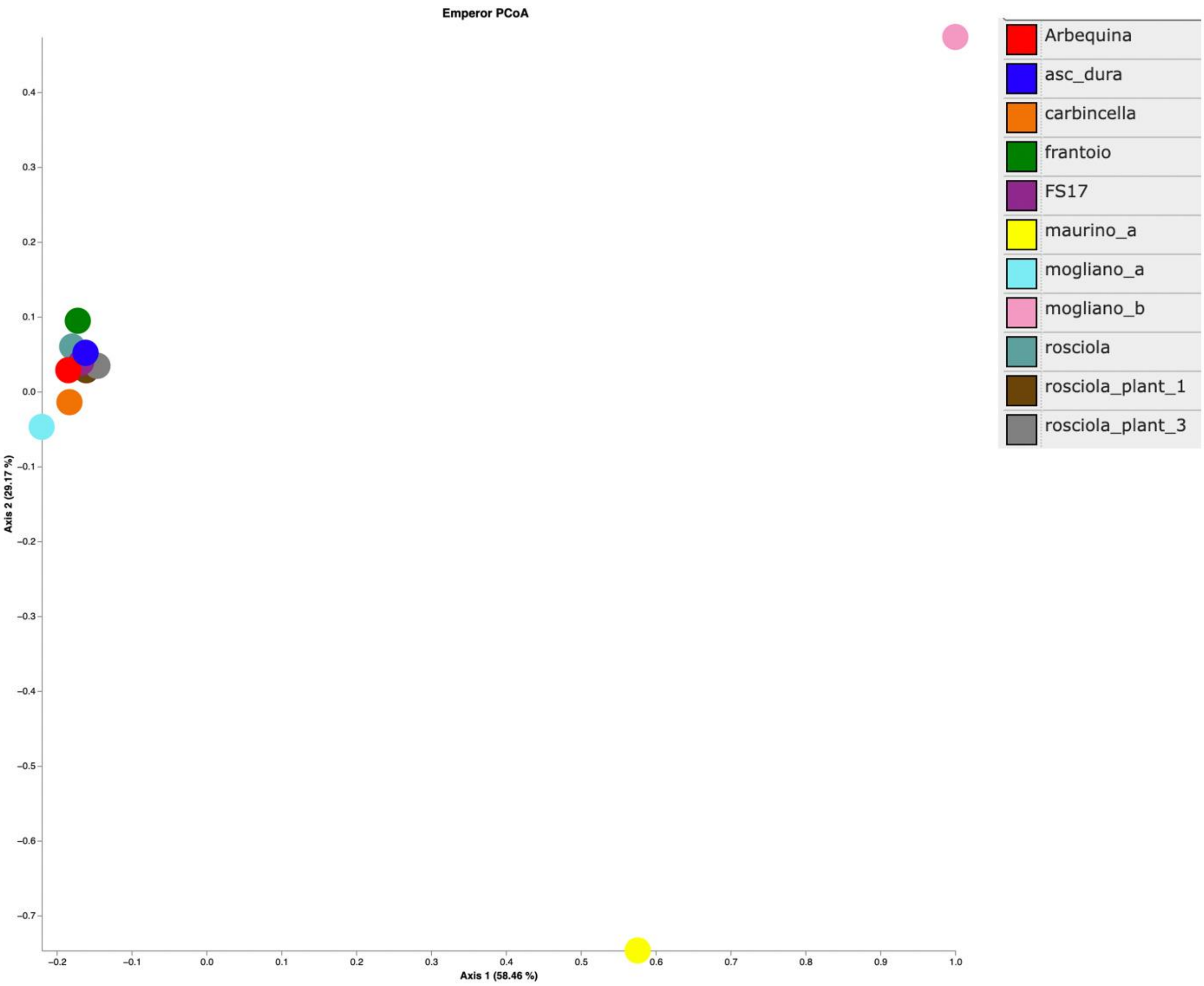

Supplement: S1 Fig — Samples are color-coded by variety. (PDF) [file pone.0289875.s002.pdf]
